# A Review of Pharmacokinetic and Pharmacodynamic Properties of Quetiapine IR and XR: Insights and Clinical Practice Implications

**DOI:** 10.7759/cureus.86258

**Published:** 2025-06-18

**Authors:** Kalpesh Joshi, Sameer Rao, Suyog Mehta

**Affiliations:** 1 Medical Affairs and Clinical Research, Sun Pharmaceutical Industries Ltd, Mumbai, IND

**Keywords:** 5-ht receptor, antipsychotic compliance, d2 receptor, extended-release, h1 receptor, optimal dose, quetiapine, sedation

## Abstract

Treatment adherence is a key consideration for patients with psychiatric illnesses. One of the strategies to improve compliance is the development of extended-release (XR) formulations. This narrative review compares the similarities, differences, and clinical implications of two formulations of quetiapine: immediate-release (IR) and XR. A comprehensive literature search of English-language clinical studies, pharmacokinetic (PK)/pharmacodynamic (PD) studies, reviews, and meta-analyses of quetiapine IR, XR was conducted using PubMed, Google Scholar, and the clinicaltrials.gov database. Case reports and animal studies were excluded. Quetiapine XR leads to a slow drug release, delays time to maximum plasma concentration (T_max_) by two to four hours, reduces peak plasma concentration (C_max_) by 13%, and lowers fluctuations in plasma concentration. Quetiapine XR should be administered once a day (OD) without/with a light meal at least four hours before bedtime. Quetiapine IR is usually administered twice a day without regard to food. Studies indicate lower rates of early sedation for quetiapine XR, which aids in the faster dose titration and achievement of higher doses. Real-world data also indicate quetiapine IR is associated with a higher prevalence of extrapyramidal side effects and higher concomitant use of anticholinergics; it appears to be preferred for lower sedative doses, for bipolar depression, and as an add-on to mood stabilisers. Quetiapine XR formulation offers an OD convenience, improves compliance, has lower rates of early sedation, and can achieve higher doses; it appears to be suitable for higher anti-manic/antipsychotic doses and as a monotherapy. These differences may have implications for careful patient selection. Long-term studies are needed for the comparison of weight gain and metabolic adverse events.

## Introduction and background

Introduction & methods

Schizophrenia, depression, and bipolar disorders contribute significantly to the global burden of disease. According to the Global Burden of Disease contributors (2019), the age-standardised prevalence of mental disorders per 100,000 people was 12,262 (95% uncertainty interval: 11,382·9-13,213·3). This occurred with a significant increase in cases, reaching 970·1 million in 2019 (48% higher than in 1990). Almost 80% of the age-standardised disease-adjusted life years (DALY), 1566.2 per 100,000 people, can be attributed to mood disorders and schizophrenia [1]. 

Second-generation antipsychotics (SGAs) have revolutionised the management of schizophrenia [2]. However, challenges related to long-term maintenance therapy remain overwhelming, and clinicians as well as patients continue to struggle. The CATIE study demonstrated that the majority of patients (64-82%) discontinued SGAs (olanzapine, quetiapine, risperidone, and ziprasidone) either due to the lack of efficacy or intolerable side effects within 18 months of treatment initiation [3]. The time to discontinuation can be as short as 3.5 months [4]. Similarly, a network meta-analysis demonstrated that more than 42,000 (80%) study participants treated with 32 antipsychotics from 226 studies discontinued treatment [5]. Even the short-term studies recorded 50% discontinuation rates [6,7]. Treatment continuation appears to be a better long-term maintenance strategy than dose reduction or discontinuation to prevent relapse [8]. A Cochrane analysis (30 randomized controlled trials (RCTs) and 4249 participants) indicated the relative risk of relapse was reduced by 62% with continued use of antipsychotics vs placebo over 7-12 months (number needed to treat for the benefit (NNTB) was 3) [9]. Thus, long-term maintenance therapy of antipsychotics is crucial. Adherence (<20% missed medication) is a key indicator for long-term treatment continuation and better clinical outcomes [10]. Non-adherence or non-compliance is associated with early treatment discontinuation, relapse, poor insight, higher substance abuse, and hostility [11]. Compliance is multi-factorial. It includes patient-related factors, such as lack of insight, or medication-related factors. Among the medication-related factors, dosage of the medication, frequency of administration, formulation, and side effects are important [12]. One of the strategies to overcome drug-related non-compliance is the development of once-a-day oral and long-acting injectable/depot formulations of antipsychotics [13].

Quetiapine XR (extended release), which is administered once daily, is one such example. Quetiapine is a commonly prescribed atypical antipsychotic, and acts on multiple receptors like H1, D1, D2, 5HT2, α-2, and muscarinic receptors [14,15]. The immediate-release (IR) quetiapine is administered twice (or rarely thrice) a day for the management of schizophrenia and bipolar disorders [16]. Usually, patients are started on a low dose and titrated slowly in several steps. However, sedation is the key side effect and may impede the upward dose titration and the achievement of the optimal dose [17]. Quetiapine XR is approved for the management of schizophrenia, bipolar disorders, and major depressive disorder (MDD) [18-19]. 

The once-a-day quetiapine XR formulation improves compliance and may lower healthcare costs. However, clinicians are often hesitant to use once-a-day formulations. There is a concern that if a single XR dose administration is missed, 100% of the daily dose is missed. With one dose of IR missed, one can receive the other dose and at least 50% of the daily dose [20]. Also, given similar indications and the bioequivalence of quetiapine IR and XR, it is difficult to appreciate the differences in the formulations. Amongst SGAs, quetiapine prescriptions appear to be increasing [21,22]. However, there is hardly any IR/XR-specific data available. Thus, revisiting the meaningful differences between IR and XR formulations and their impact on routine psychiatric practice is imperative. This narrative review aims to compare the pharmacological properties of quetiapine XR vs IR formulation and offers possible clinical implications. 

## Review

Methods

For this narrative review, we performed a literature search till February 2025 using PubMed, Google Scholar, and Clinicaltrials.gov, using the keywords "quetiapine" OR "quetiapine immediate release" AND "quetiapine XR" OR "extended release" with one of the following indications: "schizophrenia", "bipolar disorder", "anxiety disorders", and "depression". The type of articles included (by applying search filters) were "Clinical Trial", "Clinical Trial, Phase I", "Clinical Trial, Phase II", "Clinical Trial, Phase III", "Clinical Trial, Phase IV", "Randomized Controlled Trial", "review articles", "systematic reviews", "meta-analysis", and "English"-language only articles.

We also included human pharmacokinetic and pharmacodynamic studies. United States Food and Drug Administration (FDA) product labels were also accessed. Case reports, case series, consensus statements, guidelines, position statements, or animal study-related publications were excluded. Key outcome parameters of interest were pharmacokinetic, pharmacodynamic properties, dosage and administration, efficacy, and tolerability variables.

This being a narrative review, it does not cover any further statistical analyses or Inferences. The information presented is descriptive in nature and is cross-referenced wherever possible.

Results

Pharmacokinetic Properties of Quetiapine IR and XR

Table 1 [16,18,23-26] provides a comparison of pharmacokinetic properties between quetiapine XR and IR. 

**Table 1:** Comparison of pharmacokinetic properties of quetiapine XR and IR References: [16,18,23-26] C_max_: maximum plasma concentration; AUC: area under curve; XR: extended release; IR: Immediate release; T_max_: time to reach maximum plasma concentration; GMR: geometric means ratio; L: liter; T1/2: half life; BID: *bis in die* (twice a day); OD: *omne in die* (once daily)

	Quetiapine XR	Quetiapine IR	Inference
Oral absorption	Good	Bioavailability is 100% compared to the oral solution	Exact bioavailability for the XR formulation is not reported
Effect of high-fat meal	Significant increase in C_max_ (44-52%) and AUC (20-22%) respectively	A relatively small increase in C_max_ (25%) and AUC (15%).	Food leads to minimal change in bioavailability for quetiapine IR; it can be taken with/without food. Quetiapine XR is recommended to be taken without food or with a light meal.
C_max_	495.3 ng/ml (for 300 mg OD)	568.1 ng/ml (for 150 mg BID)	Reduced C_max_ (13%; ratio: 0.87; 90%CI: 0.77, 0.99) for quetiapine XR (smaller peak in plasma concentration, flatter curve)
C_min_	95.3 ng/mL	96.5 ng/mL (ratio: 1.00; 90%CI: 0.77 – 1.31)	Comparable minimum concentration
Median T_max_ (Range)	5-6 hours (0.90 – 20.0)	2 hours (0.6 – 0.8)	Delayed T_max_ (2-4 hours delayed peak in plasma concentration).
AUC (0-24)	6147 ng.h/mL	5882 ng.h/mL GMR (XR/IR): 1.04 (90% CI: 0.92-1.19)	Quetiapine AUC is similar following the administration of IR and XR formulations
Variation in daily plasma concentrations	39.2%	51.2%	Lesser variation in the plasma concentrations of quetiapine XR Vs IR
Plasma protein binding (%)	83%	83%	--
Apparent volume of distribution (L)	510 – 710	510 – 710	--
Metabolism	--	--	Less than 1% excreted unchanged. 73% is eliminated through urine and 21% through faeces.
Norquetiapine (%)	46-56%	44.8 – 54.5%	--
T1/2 (Quetiapine)	7 hours	6 hours	Appears to be the same
T1/2 (Norquetiapine)	12 hours	Not available	--

Both formulations are considered to be bioequivalent [27]. The minimum plasma concentration (C_min_), the area under the curve (AUC), bioavailability, and the half-life (T1/2) appear to be similar for both formulations. However, XR formulation prevents a sudden increase in plasma concentration by slowing the release of quetiapine [23]. Its peak plasma concentration (C_max_) is reduced by 13% (90%CI: 0.77, 0.99), time to peak plasma concentration (T_max_) is delayed by two to four hours, and has a lower percentage coefficient of variation (39.2% vs 51.2%) as compared to the IR formulation [23]. This leads to a smooth, steady-state plasma concentration by minimizing fluctuations and providing convenience with a once-a-day dosage [28]. 

Food has minimal effect on quetiapine IR; it can be taken with/without food (AUC and C_max_ increase by 15% and 25%, respectively), and the night-time dose should be administered 30-60 minutes before bedtime [16]. However, heavy/fatty meals may adversely impact the bioavailability of quetiapine XR (Increase in C_max_ (44% to 52%) and AUC (20% to 22%), respectively) and hence should be avoided with it [18]. Quetiapine XR has a median T_max_ at five to six hours, and hence, it should be administered about four to five hours before bedtime or 90 minutes before bedtime with large fatty meals. Since sedation is a key dose-limiting side effect, this strategy should help peak plasma concentrations to coincide with sleep [29].

In case of withdrawal, a gradual withdrawal is recommended for both of the formulations. The XR formulations should not be chewed, crushed, or split. Overall, both quetiapine IR and XR demonstrate linear PK properties and a predictable dose-response relationship [28].

Quetiapine is extensively metabolised and leads to the formation of two inactive metabolites and an active metabolite, norquetiapine, by the CYP3A4 pathway. Norquetiapine is further metabolised by CYP2D6. Thus, it can be susceptible to genetic polymorphism or drug-drug interactions. Old age, female sex, and poor metaboliser status of CYP2D6 can affect concentrations of norquetiapine and may lead to side effects such as sedation and anticholinergic effects [30]. Norquetiapine follows the pharmacokinetic properties of the given formulation. Similar to the parent drug, norquetiapine T_max_ was delayed (mean ± SD: 6 ± 3.3 Vs 2.1 ± 0.5 hours) and C_max_ was reduced (346 ± 110 vs 624 ± 100 nmol/L) in XR formulation as compared to the IR formulation without any significant impact on the AUC (5740 ± 2100 vs 6100 ± 1400 h/nmol/L) [31]. There are hardly any studies available to investigate the difference in the adverse events related to the norquetiapine generated by the two formulations. The C_max_-related side effects, such as orthostatic hypotension, dryness of mouth, headache, nausea, and sedation associated with norquetiapine appear to be fewer for quetiapine XR than IR [12,32,33].

Overall, treatment adherence is a key consideration in the long-term management of psychiatric conditions. The use of long-acting formulations can be one of the strategies to improve treatment compliance. There are numerous examples of XR formulations used in neuropsychiatric conditions with a ‘flat curve’ compared to IR formulations [20]. Levetiracetam XR, ropinirole XR, carbamazepine ER, valproate ER, and oxcarbazepine modified release (MR) are some examples. These formulations offer once-a-day convenience and can lead to improved adherence [34-36]. Similarly, long-acting injectable formulations of antipsychotics such as paliperidone are available [37]. This is particularly useful in patients with psychiatric conditions where the pill burden is relatively high. A study reported that non-adherence rates may range up to 56%, 50%, and 40% for patients with schizophrenia, MDD, and bipolar disorders, respectively [38]. Thus, treatment adherence is a key consideration before choosing long-term therapy.

However, there is an apprehension about the possible risk of relapse due to missed dose may be greater for XR formulations administered once daily as compared to IR formulations administered twice a day. Usually, patients will miss one dose of the medicine [20]. If one dose is missed, another IR dose may be received. At least one dose of the IR formulation (50% of the total dose) is received, even if 50% is missed. In a similar situation for XR, the total daily dosage is missed. In this case, the C_min_ at the end of missed doses appears to be similar for both quetiapine formulations. Dosing irregularities leading to worsening in the plasma concentrations were not observed with quetiapine XR as compared to the IR formulation [39]. Similarly, a computer simulation study of topiramate ER once-daily formulation indicated that there were no added risks of missed doses as compared to topiramate IR twice-a-day formulation [40]. Thus, a review of pharmacological properties and their impact on clinical practice is important. In a naturalistic retrospective study of patients with schizophrenia prescribed quetiapine IR was associated with higher rates of non-compliance vs XR formulation (12% vs 3.4%, p=0.03) [41]. Thus, preliminary evidence indicates a trend towards higher treatment adherence with quetiapine XR vs the IR formulation.

Pharmacodynamic Properties of Quetiapine IR and XR

Table 2 provides a comprehensive summary of its receptor pharmacology and subsequent clinical effects of quetiapine and norquetiapine [42-47].

**Table 2:** Dose-response relationship for quetiapine and norquetiapine * Also, H2,3 & 4, β1 and β2 receptor occupancy, effect at the clinical doses, appear negligible, and are not included in the above table for both quetiapine and norquetiapine. ** Receptor affinities were defined as: High affinity (<10nm), moderate affinities (10–100 nM); low affinities (100–1000 nM), and negligible (>1000 nm). ***The average D2 occupancy was −2%±2%from 150 mg/d; 5%±7%from 300 mg/d; 14%±11% from 450 mg/d; and 19%±1%from 600 mg/d. # No valvulopathy observed clinically. Valvulopathy was common with the 5-HT2B agonist effect, whereas quetiapine has an antagonistic effect. References: [42-47] AEs: adverse events; BDNF: brain-derived natriuretic peptide; NA: not available; NE: norepinephrine; NET: norepinephrine transporter; 5-HT: serotonin; D: dopamine; H: histamine; M: muscarinic; α – adrenergic alpha receptors; PFC: prefrontal cortex; EPS: extrapyramidal symptoms

Receptors^*^	5-HT_1A_	5-HT_2A_	5-HT_1B_,5-HT_1D_, 5-HT_1E_, 5-HT_2B_, 5-HT_2C_, 5-HT_3_, 5-HT_4_, 5-HT_5_, 5-HT_6_	5-HT_7_	D_1,2,3,4,5_	H_1_	M_1,2,3,4,5_	NET	α_1A_,α_1B_, α_2A_, α_2B_, α_2C_
Quetiapine affinity^**^	Low-moderate affinity	Low-moderate affinity	Low-moderate affinity	Low-moderate affinity	Low-moderate affinity	Moderate	Negligible	Negligible	Low-medium binding capacity
Nor-Quetiapine affinity^**^	Moderate and 10 times more potent	6 times higher affinity than quetiapine	Low-moderate affinity	Moderate and higher than quetiapine	Low-moderate affinity	High affinity and more potent than quetiapine	20-80 times more potent than quetiapine	100 times more potent and selective inhibitor	Low-medium binding capacity; more potent at α_2_
Effect on the receptor	Partial Agonist	Antagonist	Antagonist	Antagonist	Antagonist	Antagonist	Antagonist	NE reuptake inhibitor	Antagonist
Pharmacological effect	anti-depressant and anxiolytic effects	Improvement of negative and cognitive symptoms, and reduced hyperprolactinemia and extra-pyramidal symptoms	5HT_2B_ - No valvulopathy was observed clinically^#^	Antidepressant and sedative/sleep-promoting effects	Anti-psychotic Effect at higher doses, low potential for EPS	Sedation and weight gain	Anticholinergic side effects seen at overdosage - predominantly due to Norquetiapine M3: weight gain, hyperglycaemia, and antipsychotic associated diabetes	Antidepressant effect	Both can lead to orthostatic hypotension. Closely related to C_max_
Effective dose range	Intermediate doses: 300–600 mg/day			NA	High doses: 600-800 mg/day	Low doses: from 25mg onwards	Anticholinergic AEs: overdosage (4-12 gm of quetiapine), metabolic AEs: low-intermediate doses 150-300 mg	NA	NA
Receptor Occupancy	5-HT2A occupancy: 20%±2% at 150 mg/d; 57%±12% at 300 mg/d; 68%±13% at 450 mg/d; and 78%±15% at 600 mg/d.			NA	Transient: 58-64% 2-3 hours and negligible at 12 hours post-single-dose. ***	90% at 50mg	NA	19% at 150mg 35% at 300mg	NA

It can be observed from Table 2 that quetiapine and its active metabolite, norquetiapine, act on a variety of receptors. However, there are some subtle differences. Norquetiapine exhibits additional receptor pharmacology properties. It leads to norepinephrine transporter (NET) inhibition and partial agonism of 5-HT1A. These properties are not exhibited by quetiapine. Further, it has a greater affinity and produces stronger antagonism of 5-HT2A, 5-HT2C, 5-HT2B, 5-HT7, α2, H1, and muscarinic M1, M2, and M3 receptors than quetiapine. The blockade of D2/D3 receptors (antipsychotic effect) and α1 receptors (orthostatic hypotension, dizziness) appears to be similar for both quetiapine and norquetiapine. NET inhibition (35% at 300 mg/day) leads to an increase in synaptic NE concentration and improved DA release from the prefrontal cortex (PFC). Subsequently, anxiolytic and antidepressant effects are seen. This, along with a stronger partial agonism at 5HT1A receptors, norquetiapine appears to contribute to the anxiolytic and antidepressant efficacy of quetiapine, at least partly [48,49].

Quetiapine has a unique mechanism of action with a more profound effect on 5-HT receptors than D2 receptors [50]. The effect on various 5-HT receptors is profound and can be observed at mid-range doses (300-600 mg/day) [24]. 5-HT2A receptor antagonism leads to dopamine release in the mesocortical pathway and the tubero-infundibular pathway. This leads to improvement of negative and cognitive symptoms, and lower rates of hyperprolactinemia and extra-pyramidal symptoms as compared to the other antipsychotics. Norquetiapine demonstrates greater affinity than quetiapine at various 5-HT receptors. Both the parent molecule and active metabolite demonstrate a dose-response relationship in 5-HT2A receptor occupancy [49]. On the contrary, D2 receptor occupancy can be observed only at the high doses (above 600 mg/day), often transiently, peaks around C_max_, and then throughout. Both quetiapine and norquetiapine demonstrate similar and relatively low affinity to D1-5 receptors. Both dissociate relatively quickly from the receptors [27]. The mean dose of quetiapine XR required to achieve 50% D2 receptor occupancy at a steady-state concentration was estimated to be 638 mg/day. A moderate D2 receptor occupancy (65-80%) can be attributed to the fine balance between desired efficacy and minimal extrapyramidal side effects [44].

Interestingly, there is a difference in D2 receptor occupancy in IR and XR formulations. At C_max_, the D2 receptor blockade produced by quetiapine XR was lower as compared to quetiapine IR (XR: 32±11% (n=11); IR: 50±4% (n=9); p=0.0003). However, there was no significant difference observed in the D2 receptor occupancy at trough plasma concentrations (IR: 7±7% (n=10), XR: 8±6% (n=10); p=0.7). The time relationship indicated that D2 receptor occupancy peaked for quetiapine IR at two hours and then dropped. However, D2 receptor occupancy for quetiapine XR was less pronounced at C_max_ and remained at a higher level than IR throughout. Also, there was a profound fluctuation in D2 receptor occupancy for quetiapine IR as compared to XR [31]. Overall, the relationship between differential D2 receptor occupancy of quetiapine IR and XR and their effect on EPS rates remains unevaluated. Also, the dose-response relationship for D2 blockade and EPS seems non-existent for quetiapine, which is exceptional amongst antipsychotics [51].

H1 receptor antagonism appears to be one of the key indicators for side effects such as sedation, an increase in appetite, and weight gain. Sedation is a key side effect and a concern for patients as well as clinicians. Norquetiapine binds H1 receptor with high affinity [48]. However, differential H1 blockade leads to lower rates of early sedation in quetiapine XR compared to IR. It can be argued that sedation could be attributed to the C_max_. Quetiapine IR twice daily administration is associated with two such peaks in plasma concentrations, whereas XR once daily has only one relatively flat peak. Also, XR is given at night, which coincides with sleep. Further, at higher doses, quetiapine enhances synaptic norepinephrine levels by inhibiting the NET, which may counteract sedation [49]. The studies related to sedation are further elaborated in the safety section. Lastly, norquetiapine has a higher affinity for M1, M3, and M5 muscarinic receptors and H1 histaminergic receptors. It may also contribute to quetiapine's sedation, weight gain, and anticholinergic properties [29]. However, this remains to be investigated.

Comparison of Dosage, Administration, and Switching Information.

Table 3 provides comparative information on the dosage and administration of quetiapine IR and XR for their approved indications [16,18].

**Table 3:** Comparison of dosage and administration information of quetiapine IR and XR * Based on the response and tolerability may be administered three times daily ** Approved only as an adjunctive therapy with antidepressants. *** Approved in a few countries like Australia and Canada, but not in the United States/Europe. Reference: [16,18] IR: immediate release; XR: extended release; BID: *bis in die* (twice a day); TID: *ter in die* (three times a day); OD: *omne in die* (once daily)

Step	Quetiapine IR Dosage	Quetiapine XR Dosage
Schizophrenia - Adults		
Starting Dose	25 mg BID	300 mg OD
Incremental Steps	Day 2 & 3: 25-50 mg BID/TID	Day 2: 300 mg OD incremental dose
	Day 4: 300-400 mg/day	
	Further dosage increment of 25-50mg in two doses over no less than two days	
Recommended Dose	150-750 mg/day	400-800 mg/day
Maximum Dose	750 mg/day	800 mg/day
Schizophrenia - Adolescents (13-17 years)		
Starting Dose	Day 1: 25 mg twice daily (50mg total daily dose)	Day 1: 50 mg/day
Incremental Steps*	Day 2: Twice daily dosing totaling 100 mg	Day 2: 100 mg/day
	Day 3: Twice daily dosing totaling 200 mg	Day 3: 200 mg/day
	Day 4: Twice daily dosing totaling 300 mg	Day 4: 300 mg/day
	Day 5: Twice daily dosing totaling 400 mg	Day 5: 400 mg/day
	Further adjustments should be in increments no greater than 100 mg/day within the recommended dose range of 400-800 mg/day.	
Recommended Dose	400-800 mg/day	400-800 mg/day
Maximum Dose	800 mg/day	800 mg/day
Bipolar I Disorder manic or mixed - Acute monotherapy or adjunct to lithium or divalproex – Adults		
Starting Dose	Day 1: Twice daily dosing totaling 100 mg	Day 1: 300 mg/day
Incremental Steps	Day 2: Twice daily dosing totaling 200 mg.	Day 2: 600 mg/day
	Day 3: Twice daily dosing totaling 300 mg.	Day 3: between 400 and 800 mg/day
	Day 4: Twice daily dosing totaling 400 mg.	--
	Further dosage adjustments up to 800 mg/day by Day 6 should be in increments of no greater than 200 mg/day.	
Recommended Dose	400-800 mg/day	400-800 mg/day
Maximum Dose	800 mg/day	800 mg/day
Bipolar I Mania (children and adolescents 10-17 years), monotherapy		
Starting Dose	Day 1: Twice daily dosing totaling 50 mg	Day 1: 50 mg/day
Incremental Steps*	Day 2: Twice daily dosing totaling 100 mg.	Day 2: 100 mg/day
	Day 3: Twice daily dosing totaling 200 mg.	Day 3: 200 mg/day
	Day 4: Twice daily dosing totaling 300 mg.	Day 4: 300 mg/day
	Day 4: Twice daily dosing totaling 400 mg.	Day 5: 400 mg/day
	Further adjustments should be in increments no greater than 100 mg/day within the recommended dose range of 400-600 mg/day.	
Recommended Dose	400-600 mg/day	400-600 mg/day
Maximum Dose	600 mg/day	600 mg/day
Bipolar Depression-Adults		
Starting Dose (OD)	Day 1: 50 mg/day	Day 1: 50 mg/day
Incremental Steps (OD)	Day 2: 100 mg/day	Day 2: 100 mg/day
	Day 3: 200 mg/day	Day 3: 200 mg/day
	Day 4: 300 mg/day	Day 4: 300 mg/day
Recommended Dose (OD)	300 mg/day	300 mg/day
Maximum Dose (OD)	300 mg/day	300 mg/day
Major Depressive Disorder (MDD)** and Generalised Anxiety Disorder (GAD)*** - Adults		
Starting Dose	--	Day 1: 50 mg/day
Incremental Steps	--	Day 2: 50 mg/day
	--	Day 3: 150 mg/day
Recommended Dose	--	150-300 mg/day
Maximum Dose	--	300 mg/day

It can be observed that the usual starting dose, incremental doses, and maximum recommended dose for quetiapine XR are higher than quetiapine IR. Similarly, few studies indicate more use of quetiapine XR as a monotherapy and at a higher dose than quetiapine IR. In a retrospective study, significantly higher doses were achieved for quetiapine XR vs IR in patients with schizophrenia (XR: 593 mg vs IR: 338 mg; p<0.001) as well as bipolar disorders (XR: 466 mg vs IR: 308 mg; p=0.009). Similar findings were observed in other studies [25,26,32,52].

For most of the indications, quetiapine IR is administered twice or sometimes thrice daily, and quetiapine XR is administered once daily. The only exception is bipolar depression, where quetiapine IR is administered once daily at night, and the schedule is the same for both formulations. Since both formulations are bioequivalent, a switch can be done almost instantaneously. Data from the switching studies indicate that formulations can be exchanged without any meaningful impact on efficacy and tolerability. However, individual dose adjustments may be necessary [16,18]. Also, in a real-world study, patients switched from quetiapine IR to XR achieved a 21% higher mean daily dose [26]. Lastly, if patients are to be switched from quetiapine XR to IR, the psychiatrist should consider the possibility of sedation, tachycardia, and hypotension. To avoid this, a higher fraction of the total daily dose (e.g., 75 mg of 100 mg/day) can be given in the evening, one hour before bedtime, and a smaller fraction (the remaining 25 mg) can be administered in the morning. Also, the treating physician should carefully educate patients/caregivers about the change in the formulation and dosing frequency. National Health Service (NHS) bulletin (2017) recommends monitoring blood pressure in such patients [53]. In a switch-over study from quetiapine IR to XR, both efficacy and tolerability were maintained [54]. Overall, though both formulations are interchangeable, one should be careful about switching from XR to IR [18].

In a real-life scenario, the usage of quetiapine IR and XR differs. In a longitudinal real-world study of bipolar patients (n=1761), it was observed that quetiapine XR was prescribed to patients with a higher mean daily dose, those with higher psychiatric burden, younger in age, and with higher employment rates than quetiapine IR [26]. Thus, one may consider switching quetiapine IR patients with schizophrenia and bipolar mania who are stabilised on higher doses to quetiapine XR. Patients with a new onset of schizophrenia or bipolar episode, concerned about sedation and needing faster titration to achieve higher doses, or who have compliance concerns, can be initiated on quetiapine XR. On the other hand, those patients who require relatively smaller doses or can tolerate sedation (e.g., bipolar depression) can be better suited for quetiapine IR [25]. 

Efficacy Profile of Quetiapine IR and XR

Both quetiapine IR and XR are approved for the management of schizophrenia and bipolar disorder. Additionally, quetiapine XR is approved as an add-on to antidepressants in patients with MDD [16,18]. Also, quetiapine XR is approved for the management of GAD in Australia and Canada, but not in the United States/Europe [55]. The efficacy of both IR and XR formulations of quetiapine appears to be similar for the management of acute as well as long-term management of patients with schizophrenia and bipolar disorders. A review of bipolar studies (acute mania, acute depression, and maintenance therapies) indicates generally a single-digit NNT (number needed to treat) for both formulations. Similarly, NNH (number needed to harm) seems to be acceptable and similar for both formulations. Thus, it can be concluded that both formulations have equivalent efficacy and tolerability profiles. This is also further confirmed by the switching studies, which demonstrate no change in efficacy or safety. The NNT/NNH values for quetiapine IR and XR from five key publications are summarised in Table 4 [56-60]. 

**Table 4:** Comparison of NNT/NNH (95% CI) values of quetiapine IR and XR in adults with schizophrenia, bipolar mania, bipolar depression, and major depressive disorder studies * Vs placebo (95% CI) in adults with bipolar monotherapy studies/pooled analyses. For bipolar depression, either monotherapy or combination therapy was considered. ** Low- to moderate-quality evidence *** for refractory MDD. ^$^At week 4, the observed case analysis add-on setting for MADRS sustained response rate. References: [56-60] QIR: Quetiapine IR; QXR: Quetiapine XR; MADRS: Montgomery-Åsberg Depression Rating Scale; NNT: number needed to treat; NNH: number needed to harm

	Acute bipolar mania^*^	Acute bipolar depression^**^	Schizophrenia	Major depressive disorder
NNT for response rate				
QIR	6.0 (3.8 to 13.5)	300 mg: 5.6 (4.7 to 6.9)	20 (13 to 53)	--
QXR	4.6 (3.4 to 7.2)	4.5 (3.3 to 6.9)	8 (6 – 11)	150 mg: 12 300mg: 10^***^
NNT for remission rate				
QIR	6.9 (4.3 to 17.9)	300 mg: 5.3 (4.5 to 6.4)	NA	NA
QXR	7.2 (4.5 to 16.3)	6.8 (4.3 to 15.6)	NA	NA
NNH for discontinuation due to AEs				
QIR	144.7 (41.0 to 25.8)	300 mg: 24.6 (16.3 to 49.5); 600 mg: 11.4 (8.9 to 15.7)	--	--
QXR	20.6 (44.5 to 13.6)	9.1 (6.1–18.3)	400 mg/day: 36 (37 to 11)	9 (15 to 6)^$^
			600 mg/day: 889 (21 to 19)	
			800 mg/day: 1586 (20 to 21)	
NNH for somnolence/sedation				
QIR	--	300 mg/day: 6 (5 to 8)	9 (7 to 13)	--
		600 mg/day: 7 (5 to 9)		
QXR	400-800 mg/day: 8 (18 to 5)	300 mg/day: 4 (7 to 3)	600 mg/day: 15 (112 to 8)	300 mg/day: 5 (4 to 6)

In the real-life scenario, however, it was observed that quetiapine XR was more likely to be used in higher doses in newly diagnosed patients with schizophrenia. On the contrary, quetiapine IR was used in lower doses and more often as an add-on for additional sedative, anxiolytic, or antidepressant features [25,33,46,61].

Safety Profile of Quetiapine XR and IR

**Figure 1:**
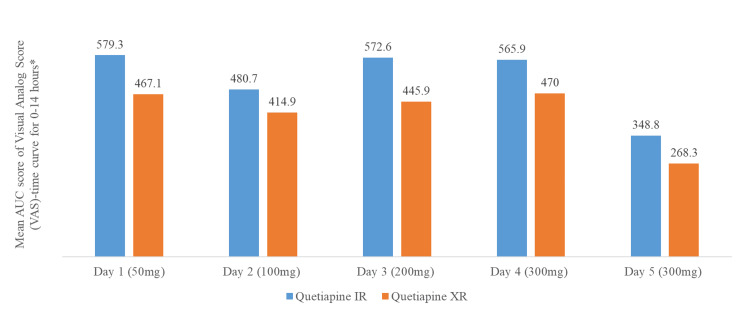
Sedation profile of quetiapine IR and XR over the first five days of administration (n=58, healthy volunteers)* * Except for day 5 where it was done for 0-11 hours. P value for the comparison between quetiapine IR and XR, day 1 = 0.004, day 2 = 0.067, day 3 <0.001, day 4 = 0.029, and day 5 = 0.001 Reference: [17] IR: immediate release; XR: extended release; AUC: area under the curve

Few studies indicated a meaningful difference in the sedation for quetiapine XR vs quetiapine IR. Early sedation appears less with the XR formulation than with the IR formulation [62]. Similarly, in a crossover, healthy volunteer study comparing quetiapine IR and XR (n=58), the peak sedation appeared less with the quetiapine XR group. The numerical difference was maintained up to eight hours post-dose. This pattern was observed over five days of administration (Figure 1) [17]. However, long-term studies are missing in this regard. 

Another finding was that the total number of adverse events was numerically higher in the patients who received quetiapine IR (21.7%) vs. quetiapine XR (9.8%). Dryness of mouth, dizziness, headache, nausea, abnormal dreams, nasal congestion, and dysarthria were more common in the quetiapine IR group [17].

H1 antagonism appears to be the most common mechanism for antipsychotic-associated weight gain [63]. In a meta-analysis of RCTs of acute schizophrenia with a median treatment duration of six weeks, quetiapine was associated with a weight gain of 1.56 kg (95%CI: 1.09-2.04), which appears to be moderate weight gain as compared to weight gain associated with olanzapine 2.73 kg (95%CI: 2.38-3.07) and clozapine 3.01 kg (95%CI: 1.78-4.24) [64]. Both formulations appear to be associated with weight gain. Similar inferences can be applied to other parameters such as blood glucose levels, BMI change, serum cholesterol levels, and precipitation rate of type 2 diabetes. The difference in the two formulations appears to be minimal [65]. However, no head-to-head studies are available comparing the effects of the two formulations on the metabolic parameters. Physicians should carefully monitor the patients, especially quetiapine XR, since it seems to be used in higher doses.

Extrapyramidal reactions are key considerations for the choice of antipsychotics. We assessed the impact of formulations on the extrapyramidal symptoms (EPS). There are very few comparative data for EPS, but in one of the studies assessing the tolerability, quetiapine XR was associated with a lower prevalence (2.9% vs. 11.6%), lower severity of EPS, and fewer patients reporting worsening of EPS (4.3% vs. 13%) than quetiapine IR on the Simpson-Angus Scale [66]. A study in Taiwanese patients with schizophrenia assessing the tolerability of quetiapine XR when switched from other antipsychotics, including quetiapine IR, found that only one patient developed akathisia, and use of anticholinergic drugs decreased from 15% to 8.3% [67]. However, switching studies failed to demonstrate any difference in the EPS profile [68,69]. Thus, while there is evidence that formulations may impact EPS differently, conclusive evidence from robust studies is still lacking. 

There are few studies done to assess the difference in overdosage and toxicity for the two formulations of quetiapine. A retrospective study (n=256) indicated a requirement of larger mean dose (5.7g for XR vs 1.75g for IR; P = 0.004), delayed onset of overdosage symptoms, and delayed time to recovery for quetiapine XR as compared to the IR [70]. Co-medications such as benzodiazepines, other psychotropic drugs, and antidepressants were common and often increased the severity of quetiapine toxicity [71]. There are a few reports of addiction liability with quetiapine, especially the IR formulation, due to the fast onset of PK properties and sedative effect [24]. Lastly, tolerance may be developed for the short-lived sedative effect; however, it has not been reported with other effects of quetiapine. 

Comparison of Quetiapine IR and XR

Table 5 compares the overall properties of quetiapine IR and XR [18,20,24].

**Table 5:** Comparison of quetiapine IR and XR References: [18,20,24] IR: immediate release; XR: extended release; T_max_: time to maximum plasma concentration; C_max_: peak plasma concentration

Quetiapine XR	Quetiapine IR
Once-a-day dosage, reduced pill burden, and probability of improved compliance.	Twice a day
Delayed T_max_ by 2-4 hours. Peak plasma concentrations (C_max_) were reduced (by 13%) Vs IR formulation.	Higher fluctuations in the plasma concentration in a day.
Food delays C_max_ & heavy meals should be avoided.	No impact of food.
Reduced early sedation; should be taken 4 – 5 hours before bedtime	Should be taken 30-60 minutes before bedtime.
Relatively simple and quick dose titration to optimal dose, efficacious dose achieved in fewer dose titration steps	Slower dose titration and needs relatively more steps of titration to achieve efficacious dose.
Higher doses achieved for quetiapine XR and hence more commonly used in schizophrenia and bipolar mania.	Better suited for lower doses. Quetiapine IR is used more commonly as an add-on therapy for bipolar depression.
More use as a monotherapy & in younger patients and less use in older, those with substance abuse disorder.	More commonly used as an add-on for the management of patients with bipolar depression.
Reduced peak D_2_ receptor occupancy & possibility of extrapyramidal side effects.	More use of concomitant anticholinergics.

From Table 5, we can see that quetiapine XR is associated with a higher starting dose, faster titration, once-daily convenience, and has some indications towards higher compliance and lower EPS. The real-world studies indicate the use of quetiapine XR in schizophrenia and bipolar mania, whereas quetiapine IR is used in patients with bipolar depression. While both formulations are bioequivalent and a dose-to-dose switch is possible, one should adopt a cautious approach. This information may help busy clinicians not only to distinguish the properties of two formulations but also for better clinical management. Further research, especially comparing the long-term metabolic adverse events such as weight gain, hyperlipidaemia, hyperglycaemia, or precipitation of type 2 diabetes, as well as the role of these formulations in the practice of precision medicine, is warranted.

## Conclusions

In long-term psychiatric conditions, one of the first important considerations is treatment adherence. XR formulations have contributed to the pharmacotherapy of diseases by delaying drug release, reducing adverse events by flattening the C_max_, and potentially improving treatment adherence. Preliminary evidence indicates a trend towards higher treatment adherence with quetiapine XR vs the IR formulation. Dosage and administration information is the second important consideration. Most of the pharmacokinetic properties appear similar for both formulations, with few notable exceptions in T_max_, C_max_, and variability in plasma concentrations. Quetiapine IR should be administered one to two hours before bedtime with/without fatty meals. However, quetiapine XR should be administered four to five hours before bedtime without fatty meals.

The third important consideration is the dose-response relationship and achievement of the optimal dosage. Quetiapine XR has a higher initial starting dose, higher incremental steps, and higher maximum dose recommended for a given condition. This is important since quetiapine demonstrates a strong dose-response relationship. This, along with once daily administration, reduced pill burden and ease of administration for quetiapine XR is a surrogate for better outcomes, especially for patients with schizophrenia and bipolar mania. The fourth important consideration is sedation, which correlates primarily to peak H1 receptor antagonism. Often, the sedation associated with quetiapine is short-lived, which may hinder the dose-up titration. Early sedation appears to be less with quetiapine XR compared to IR since C_max_ is reduced.

The fifth consideration can be other side effects. There is an early indication towards reduced EPS and overall requirement of anticholinergic drugs for quetiapine XR as compared to IR. Addiction liability appears to be present for quetiapine IR, whereas there is hardly any such information available for quetiapine XR. Lastly, long-term and head-to-head comparative data for weight gain and metabolic side effects of quetiapine IR and XR are missing. Overall, quetiapine XR has efficacy similar to quetiapine IR with the potential benefits of a once-a-day administration, improved compliance, and reduced early sedation compared to quetiapine IR. For patients with higher dose requirements, faster titration and lesser sedation, XR may be better suited. For patients with lower dose requirements and a need for sedation, IR may be better suited. These differences can be meaningful and can lead to significant improvement in a patient chosen appropriately.
